# MR imaging features of spinal pilocytic astrocytoma

**DOI:** 10.1186/s12880-018-0296-y

**Published:** 2019-01-14

**Authors:** De-jun She, Yi-ping Lu, Ji Xiong, Dao-ying Geng, Bo Yin

**Affiliations:** 1 Department of Radiology, Huashan Hospital, Fudan University, 12 Middle Wulumuqi Road, Jingan District, Shanghai, China; 2 Institute of Functional and Molecular Medical Imaging, Fudan University, 12 Middle Wulumuqi Road, Shanghai, China; 3 Department of Pathology, Huashan Hospital, Fudan University, 12 Middle Wulumuqi Road, Jingan District, Shanghai, China

**Keywords:** Pilocytic astrocytoma, Spinal cord, Magnetic resonance imaging, Diffusion-weighted imaging

## Abstract

**Background:**

The purpose of this retrospective review is to determine the MR imaging features of pilocytic astrocytoma (PA) in the spinal cord to help neuroradiologists preoperatively differentiate PA from other intramedullary tumors.

**Methods:**

Neuro-oncology database review revealed 13 consecutive patients with a pathological spinal PA diagnosis and availability of preoperative MR imaging. Three patients had preoperative diffusion-weighted MR imaging. Demographics and conventional and diffusion MR imaging records were retrospectively evaluated.

**Results:**

Among 13 cases of spinal PA, six PAs were located in the cervical region, 4 in the cervical-thoracic region, and 3 in the thoracic region. The average length of vertebral segments involved for the tumors were 4.7 ± 4.6 segments. Six tumors had associated syringomyelia. Eight PAs were located eccentrically in the spinal cord, and eleven had well-defined margins. Eight tumors (61.5%) were intermixed cystic and solid. All were contrast-enhanced, and 53.8% of all PAs showed focal nodule enhancement of the solid components. Two PAs showed intratumoral hemorrhages, and only one demonstrated cap sign. The ADC values (*n* = 3) of the tumors were 1.40 ± 0.28 × 10^− 3^ mm^2^/s (min–max: 1.17–1.71 × 10^− 3^ mm^2^/s).

**Conclusions:**

PA should be considered in the differential diagnosis of intramedullary tumors that occur in the cervical and thoracic regions. Eccentric growth pattern, well-defined margin, intermixed cystic and solid appearance, focal nodular enhancement of solid components and syringomyelia are relatively frequent features. Relatively high ADC values compared with normal-appearing spinal cord parenchyma are common in spinal PA.

## Background

Pilocytic astrocytoma (PA) is defined as grade I tumor that most frequently occurs in the cerebellum according to the latest World Health Organization (WHO) classification system [1]. PA in the spinal cord is uncommon, comprising only approximately 2–5.2% of all PAs [2, 3]. According to data from the CBTRUS, spinal PA accounts for 12.4% of primary spinal cord tumors in children and adolescents (age 0–19 years) and 0.8% in adults (age 20+ years) [4]. Despite its rarity, spinal PA has received much attention due to the significant neurological deficits it causes, typically related to its intramedullary location compared with other PA in other locations of the neuraxis. As a benign and circumscribed astrocytoma, spinal PA rarely invades the surrounding tissue, leads to malignant progression or recurs with complete surgical resection when comparing with other spinal astrocytoma of infiltrative nature. Spinal PA usually represents a clear margin between the tumor and normal spinal cord parenchyma, and therefore, a gross total resection is possible in 50–81% of the tumors [5, 6]. In addition, spinal PA commonly shows cystic components that are frequently not resected surgically [7]. Therefore, preoperative accurate diagnosis of spinal PA may be useful in the surgical planning and prognosis prediction. Due to the rarity of spinal PA, most of the imaging studies describing this disease are case reports, small case series or literature reviews [7–12]. The purpose of this retrospective study was to analyze MR imaging characteristics that may be helpful in correctly diagnosing spinal PA preoperatively.

## Methods

### Patients

By searching the neuro-oncology database of our hospital between January 2008 and February 2018, 30 consecutive patients were identified for a histopathology-proven spinal PA. Of these 30 patients, 17 patients who did not undergo MR examinations were excluded. Finally, a series of 13 patients with spinal PA (8 men and 5 women, average age at diagnosis, 31.6 years; age range, 6–65 years) were enrolled. One patient had postoperative recurrence. The clinical information, imaging and pathologic findings of these patients were reviewed.

All surgical specimens were fixed in 10% neutral-buffered formalin and stained with hematoxylin and eosin. Histopathologic diagnoses were confirmed according to the latest WHO classification of central nervous system tumors by an experienced pathologist with 8 years of experience (J.X.).

### Imaging technique and evaluation

All patients underwent magnetic resonance imaging (MRI) examinations. MR examinations were acquired using a GE 3.0-T system (GE Medical Systems) with a MR phased array spine coil. The routine imaging sequences included sagittal and axial pre-contrast T1-weighted images (T1WI), sagittal and axial T2-weighted images (T2WI), contrast-enhanced sagittal and axial T1WI and diffusion-weighted images (DWI, single shot, spin-echo, echo-planar imaging sequences), with b-values of 0 and 1000 s/mm^2^ in three orthogonal directions. The contrast enhanced T1WI were obtained after the intravenous injection of a standard dose of 0.1 mmol/kg of gadobenate dimeglumine (MultiHance, Bracco).

All MRI images were retrospectively analyzed in consensus by a fourth-year radiology resident (L.L.) and an experienced neuroradiologist with 10 years of experience (B.Y.) on a PACS workstation monitor. The following imaging findings were recorded: (a) longitudinal location of tumor, defined as the major portion of tumoral involvement among the cervical, thoracic, and lumbar vertebra regions; (b) axial location of tumor (eccentric and central); (c) lesion size, measured in the number of vertebral segments involved; (d) signal intensity on T1WI and T2WI; (e) enhancement characteristics, classified into the following categories based on portions of solid mass enhancement and homogeneity: no enhancement, focal nodular enhancement, diffuse heterogeneous enhancement (heterogeneous enhancement of one-half or more of the solid components of the lesion), and diffuse homogeneous enhancement (homogeneous enhancement of one-half or more of the solid components of the lesion) and patchy enhancement (enhancement of less than one-half of the solid component of the lesion); (f) imaging pattern (intermixture cystic and solid, solid with no cyst, and predominantly cystic); (g) presence of syringomyelia, defined as a cystic dilatation of the central area of the spinal cord, without any contrast-enhancement in the wall; (h) intratumoral hemorrhage, defined as hyperintensity on pre-contrast T1WI and hypointensity on T2WI in the tumor; (i) tumor boundary (well-defined or ill-defined); and (j) cap sign, defined as a rim of extreme hypointense signal seen at the distal or proximal pole of the tumor on T2WI, indicating depositions of hemosiderin [13–15]. When diffusion MR imaging was available, a maximum of 3 region of interests (ROI) depending on tumor size (range 1–3, size 10–20 mm^2^) were placed in the contrast-enhanced solid components of the tumors on the apparent diffusion coefficient (ADC) map. In addition, a single ROI was positioned on the normal-appearing spinal cord parenchyma.

## Results

### Patient population

The clinical and radiological features of spinal PA are summarized in Table 1. The average age at diagnosis was 31.6 years of age (range, 6–65 years) with 76.9% older than 19 years of age. Eight (61.5%) patients were male and 5 (38.5%) were female. The course of the disease ranged from 2 months to 5 years. The presenting symptoms were progressive upper limb numbness (*n* = 4), progressive bilateral lower limb weakness (*n* = 3), bilateral lower limb weakness plus bladder dysfunction (*n* = 1), unstable gait (n = 1), neck and back pain (n = 3), and sensory disturbance (n = 1). Seven patients with PA underwent gross total resection, 5 underwent subtotal resection and 1 underwent biopsy only. No patients received postoperative adjuvant radiotherapy or chemotherapy. The mean follow-up was 3.14 years (range, 0.33–7.42 years). One patient died, yielding an overall survival of 76.9% to date and a 5-year survival rate of 80.0%.

**Table 1:** Clinical and neuroradiological manifestations of 13 spinal cord pilocytic astrocytoma

Case/ gender/age (years)	Location/distribution	Vertebral segment	boundary	Signal intensity of solid tumor on T1WI/T2WI	Enhancement characteristics	Imaging pattern	Syringomyelia	Hemorrhage/cap sign	ADC value (10^−3^ mm^2^/s)	Treatment/prognosis
1/F/21	Cervical-thoracic/central	17	Well-defined	Low/high	Focal nodule	intermixture cystic and solid	+	−/−		STR/alive
2/M/7	Thoracic/central	4	Well-defined	Low/high	Focal nodule	intermixture cystic and solid	+	−/−		GTR/alive
3/M/30	Cervical/eccentric	2	Well-defined	Iso/iso	Patchy	solid with no cyst	–	+/−	1.32	STR/alive
4/F/45	Cervical/eccentric	1	Well-defined	Low/high	Focal nodule	intermixture cystic and solid	–	−/−	1.17	STR/alive
5/M/6	Cervical-thoracic /eccentric	10	Well-defined	Low/high	Focal nodule	intermixture cystic and solid	+	−/−		GTR/alive
6/M/22	Cervical-thoracic /central	9	Well-defined	Low/high	Diffuse heterogeneous	intermixture cystic and solid	+	−/−		GTR/alive
7/M/54	Cervical/eccentric	1	Well-defined	Low/high	Diffuse homogeneous	solid with no cyst	–	−/−		GTR/alive
8/M/65	Cervical/central	3	Well-defined	High/low	Focal nodule	predominantly cystic	–	+/+		GTR/alive
9/F/11	Cervical/eccentric	3	Well-defined	Low/high	Patchy	solid with no cyst	–	−/−		STR/alive
10/M/43	Cervical-thoracic /eccentric	5	Ill-defined	low/high	Diffuse homogeneous	intermixture cystic and solid	+	−/−		GTR/alive
11/F/40	Thoracic/eccentric	2	Well-defined	low/high	Focal nodule	intermixture cystic and solid	+	−/−		GTR/alive
12/F/41	Cervical/eccentric	2	Well-defined	iso/iso	Focal nodule	intermixture cystic and solid	–	−/−	1.71	STR/alive
13/M/26	Thoracic/central	2	Ill-defined	iso/high	Patchy	solid with no cyst	–	−/−		Biopsy/Died

Note: *STR* Subtotal resection, *GTR* Gross total resection

### Imaging

**Fig. 1:**
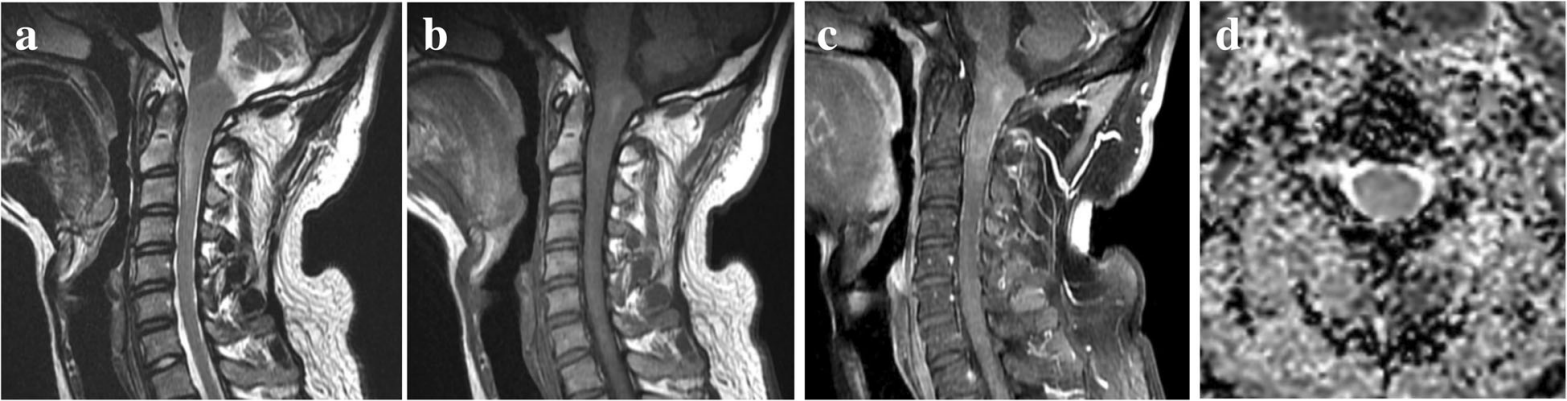
Cervical PA. **a**, Preoperative sagittal T2-weighted MR image shows a solid lesion in the cervical region presenting slightly hyperintense signal. Mild peritumoral edema is seen. **b**, Sagittal T1-weighted MR image shows a high signal intensity region corresponding to hemorrhage in the tumor. **c**, Sagittal contrast-enhanced T1-weighted MR image shows patchy enhancement of the tumor. **d**, ADC map shows the solid component shows increased water diffusion (ADC value = 1.32 × 10^− 3^ mm^2^ /s)

**Fig. 2:**
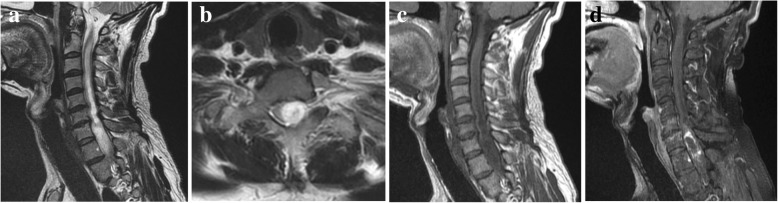
Cervical-thoracic PA. **a**, Preoperative sagittal T2-weighted MR image shows a hyperintense lesion in the cervical-thoracic spinal cord with syringohydromyelia. **b**, Axial T2-weighted MR image shows an eccentric growth pattern. **c**, Sagittal T1-weighted MR image shows the tumor at hypointense signal intensity. **d**, Sagittal contrast-enhanced T1-weighted MR image shows diffuse non-homogeneous enhancement of the tumor

**Fig. 3:**
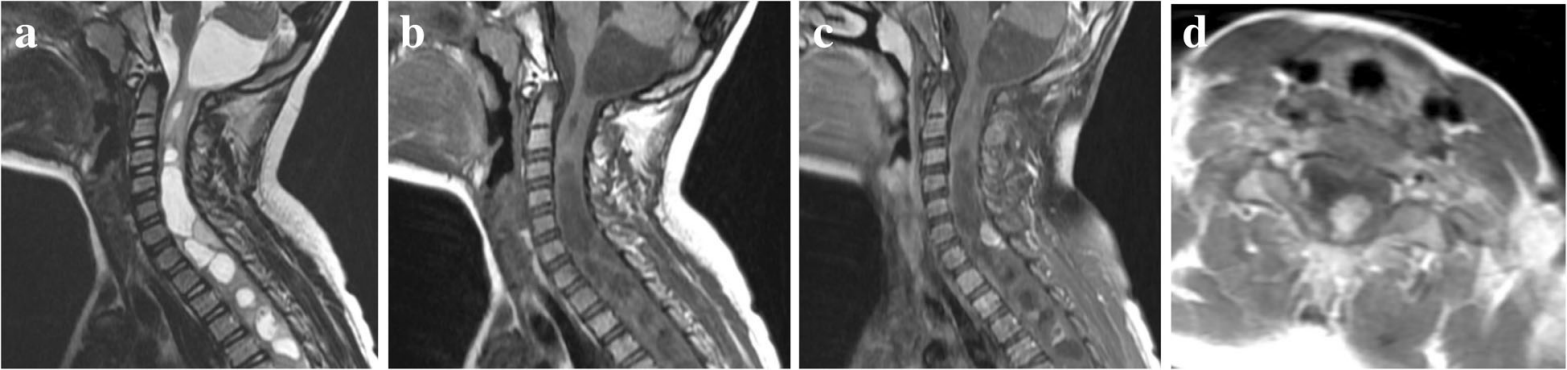
Cervical-thoracic PA. **a**, Preoperative sagittal T2-weighted MR image shows a solid-cystic lesion with multi-segmental involved of spinal cord. **b**, Sagittal T1-weighted MR image shows the solid component of lesion at hypointense signal intensity. **c**, Sagittal contrast-enhanced T1-weighted MR image shows focal nodular enhancement of solid component. **d**, Axial contrast-enhanced T1-weighted MR image shows that the solid nodule is obviously enhanced. The cystic wall demonstrates no enhancement

Of 13 spinal PAs, 6 tumors (46.15%) were located in the cervical region (Fig. 1), 4 in the cervical-thoracic region, and 3 in the thoracic region. One tumor grew along the cervical region, invading the brain stem. Concerning tumor distribution on axial images, 8 PAs (61.5%) were eccentrically located in the spinal cord (Fig. 2) and 5 (38.5%) were central. The average length of vertebral segments involved for these spinal PAs were 4.7 ± 4.6 segments (range, 1–17 vertebral segments). All but two tumors had relatively well-circumscribed margins. On the preoperative examinations, 8 of 13 (61.5%) were intermixed cystic and solid (Fig. 3). Syringomyelia was evident in six cases (46.2%). The solid portions of tumors were moderately hyperintense on T2WI and hypointense on T1WI. The cystic portions were marked hyperintense on T2WI and hypointense on T1WI. On the contrast-enhanced MR images, 7 of 13 (53.8%) PAs showed focal nodule enhancement of the solid components of tumor (Fig. 3). In addition, intense enhancement of solid components of tumors was found in 10 of the 13 PAs (76.9%), whereas mild enhancement was only found in remaining 3 cases. Two spinal PAs (15.4%) showed evidence of hemorrhage (Fig. 1), and only one case (7.7%) demonstrated hemorrhage with cap sign.

On DWI, the signal intensity of the solid parts of the tumors was isointense in 3 tumors. ADC values of solid components of tumors were measured in 3 cases. The minimum tumoral ADC values for spinal PA and normal-appearing spine cord were 1.40 ± 0.28 × 10^− 3^ mm^2^ /s (min–max: 1.17–1.71 × 10^− 3^ mm^2^ /s) and 0.79 ± 0.05 × 10^− 3^ mm^2^ /s (min–max: 0.73–0.83 × 10^− 3^ mm^2^ /s), respectively (Fig. 1). The ADC values of spinal PA were higher than those of normal-appearing spinal cord, but there were no significant differences (*P* = 0.05).

## Discussion

In this study, we report the preoperative MR imaging findings of spinal PA and describe the diffusion MR imaging metrics of these rare tumors. Although PA occurs most commonly in children and adolescents younger than 20 years, it can be encountered in all age groups ranging from infancy to the eighth decade of life [2, 3, 8]. Kiernan et al. [16] reported in a large retrospective review of all spinal astrocytomas that a mean age at presentation of spinal PA was 31.9 years with 61% of patients being male, unlike those in the cerebellum. Among 13 patients with spinal PA in the current study, the mean age was 31.6 years and the male/female ratio was 8:5; this age and sex distribution was consistent with those in previous studies [16].

Spinal PAs are relatively benign tumors characterized by slow growth and late clinical presentation. In one large series in which presenting symptoms were known, the most common symptoms of spinal cord astrocytoma were pain and sensory deficit, followed by motor deficit, which were often of long duration (average 3 years) [5]. Our cohort showed similarities with previous study with pain and sensory deficit (61.5%) being the most common presentation of spinal PA.

According to the 2016 WHO classification of central nervous system, PA represents grade I tumors histologically [1]. Histologically, PA is a benign tumor of low to moderate cellularity with loosely textured areas, consisting of multipolar cells, microcapsules, and eosinophilic granular bodies, as well as densely fibrillated areas rich in Rosenthal fibers, composed of cells with long bipolar processes and elongated cytologically bland nuclei [17]. Surgery with gross total resection is the preferred treatment and postoperative radiotherapy is not recommended for routine treatment of PA [16]. Spinal PA is remarkable for a favorable outcome with 5- and 10-year overall survival rates of 82–86% and 74–78%, respectively [16, 18]. The overall survival rates in our series are comparable.

MRI is the preferred imaging tool for detecting and characterizing spinal cord tumors, especially for intramedullary tumors [19, 20]. Although there are imaging reports of PA located in the brain [21–23], dedicated imaging studies of intramedullary PA are rare [7–12]. With respect to longitudinal location of spinal PA, 6 tumors in this series were in the cervical region, 4 in the cervical-thoracic region, and 3 in the thoracic region. This location distribution is inconsistent with the findings of Bloomer et al. [19], who found that most astrocytomas reside in the thoracic spine. However, like us, Seo et al. [24] also reported that the cervical region was the most common site of intramedullary astrocytoma. Because PA usually originate from spinal cord parenchyma and not from the central canal, it has been reported that they tend to arise eccentrically within the posterior cord [25, 26]. In current study, 76.9% PAs were eccentric type, similar to those in previous reports [7]. This axial location of PA differed from ependymoma, which show a tendency toward a central location with a reported frequency of 91.7–100% [13, 27]. In addition, most spinal PA in our study showed a well-defined margin which was similar to that of other PA in other parts of the neuraxis [23], due to its tendency to displace rather than infiltrate neural tissue. In the current study, a mean size of 4.7 vertebral segments for spinal PA was smaller than previous report of a mean size of 7 vertebral segments for astrocytoma [14].

On MRI, the predominant image pattern of PA was a cystic mass with a mural nodule; the solid component of spinal PA is hypointense on T1WI and hyperintense on T2WI [7]. Several studies demonstrated that most PA cyst walls do not enhance while some may enhance intensely [28, 29]. Spinal PA in our cohort had great propensity for cyst formation, comprising 61.5% of cases. Although there is no comparable neuroradiologic literature regarding cysts formation within spinal PA, there is evidence that cerebellum PA have a tendency for cyst formation, with an occurrence rate ranging from 44 to 86.7% [30, 31]. Therefore, our results demonstrated that PA in the spinal cord may present similar intermixed cystic and solid appearance as its intracranial counterparts.

Cerebellum PA are known to enhance, typically intensely [23]. The contrast enhancement of PA is thought to be due to the unique vascular wall of the tumor, in which endothelial cells have open tight junctions and fenestrae that allow contrast material extravasation. The contrast enhancement of spinal PA has been described in two small series studies. Crawford et al. [32] described the preoperative MR imaging findings in 6 spinal PAs of a publication of pediatric spinal cord tumors. The enhancement pattern of spinal PA varied from focal nodular, irregular, no significant enhancement to ring-enhancement. Seo et al. [24] reported that all three PA tumors in their series showed focal or diffuse contrast enhancement. However, a rare case of a non-enhancing spinal pilocytic astrocytoma has been reported in the literature [12]. In this study, all 13 PAs were contrast-enhanced after contrast administration, which is consistent with a previous study [24]. Furthermore, we found that focal nodular enhancement, as the predominant contrast-enhancement style, was observed in 53.8% of cases of spinal PA in our series.

Syringomyelia, which refers to ependyma-lined cystic central canal dilatation, is recognized as a common feature of intramedullary spinal cord tumors [13, 19, 20]. Radiologically, these cystic changes are usually located in the rostral and caudal pole of the tumors and do not show any enhancement on contrast-enhanced MR imaging. Clinically, these cysts do not need treatment and commonly resolve after tumor resection. The frequency of syringomyelia in our series of spinal PA is higher than that reported for other intramedullary astrocytomas [13].

Hemorrhage in PA has been described as an uncommon imaging feature that was observed in 2/13 (15.4%) tumors in our series. According to a previous report, there was only one spinal PA showing spontaneous hemorrhage in a literature review of spinal PA [7]. In a systematic review of PA with an emphasis on hemorrhage, White et al. [33] reported that intratumoral hemorrhage was found in 8% of cases, a greater frequency than previously thought. Degenerative mural hyalinization of vessels, thin-walled ectatic blood vessels, and dysplastic capillary beds have been speculated as potential causes for hemorrhage in PA [33]. Although hemorrhage does not seem to be a typical presentation, its presence should not exclude the diagnosis of PA. In addition, only one hemorrhage associated with PA seen in the current study was demonstrated as cap sign. Previous studies have demonstrated that a cap sign with hemorrhage, as a characteristic feature of spinal ependymomas, was useful for differentiating intramedullary ependymomas from astrocytomas [13, 14, 19]. The frequency of cap sign in spinal PA in current study is similar to that of spinal astrocytoma and lower than that of spinal ependymoma reported previously [13].

Diffusion-weighted MR imaging has been used in preoperative characterization of spinal lesions [34]. To our knowledge, only a few case reports of diffusion MR imaging characteristics of spinal PA have been reported previously [7, 35]. Maria et al. [35] reported one case of cervical PA had an increase in the ADC value (1.63 × 10^− 3^ mm^2^ /s). In the three tumors in which ADC values could be reliably measured, we found that the minimum ADC values were 1.40 ± 0.28 × 10^− 3^ mm^2^/s, which tended to higher than normal-appearing spinal cord parenchyma. These ADC values of spinal PA are comparable to those of other PA in the brain, and higher than those of ependymomas [36] and high-grade gliomas [30].

Spinal PA should be differentiated from other spinal tumors, including ependymoma, hemangioblastoma, and other spinal astrocytoma. Spinal ependymoma usually occur in lower thoracic and lumbar region, showing a central location of spinal cord [13]. Ependymoma can often display intratumoral hemorrhage, presenting a rim of extreme hypointensity seen at the pole of the tumor on T2WI due to subsequent hemosiderin (cap sign) [27]. In addition, syringohydromyelia is more common in ependymoma whereas contrast enhancement is more heterogeneous than PA [7, 13]. Hemangioblastoma has the imaging features resembling PA, but the signal void within and around the tumor on MRI is useful for differential diagnosis [14]. Compared with spinal PA, other spinal astrocytoma such as diffuse astrocytoma and anaplastic astrocytoma has an infiltrative nature with poorly defined margin [37].

Our study had some limitations. First, this was a retrospective study without a control group. Second, the number of patients was limited; however, spinal PA is an uncommon tumor, and ours is one of the largest series to date. Third, we had only three patients with PA in whom diffusion-weighted imaging was available. Therefore, conclusions based on the diffusion-weighted imaging study should be interpreted with caution. Future larger studies with emphasis on diffusion-weighted imaging of spinal PA may be of benefit.

## Conclusion

In summary, although spinal PA is rare, it should be considered in the differential diagnosis of spinal cord tumors with intermixed cystic and solid appearance associated with eccentric growth patterns, well-defined margins and focal nodular enhancement after contrast administration. Syringomyelia appears to be a common observation. Relatively high ADC values of the solid portions may distinguish these tumors from high-grade gliomas and ependymomas.

## Abbreviations

ADC: Apparent diffusion coefficient
DWI: Diffusion weighted imaging
MR: Magnetic resonance imaging
PA: Pilocytic astrocytoma
ROI: Region of interest
T1WI: T1-weighted images
T2WI: T2-weighted images
WHO: World Health Organization
